# miR‐1 suppresses the proliferation and promotes the apoptosis of esophageal carcinoma cells by targeting Src

**DOI:** 10.1002/cam4.1214

**Published:** 2017-10-16

**Authors:** Zhicong Liao, Xiaojun Wang, Hongwei Liang, Ao Yu, Uzair ur Rehman, Qian Fan, Yue Hu, Chen Wang, Zhen Zhou, Tao Wang

**Affiliations:** ^1^ Department of Thoracic and Cardiovascular Surgery Nanjing Drum Tower Hospital the Affiliated Hospital of Nanjing University Medical School Nanjing Jiangsu 210008 China; ^2^ Nanjing Medical University Affiliated Cancer Hospital Nanjing Jiangsu 210009 China; ^3^ State Key Laboratory of Pharmaceutical Biotechnology Nanjing Advanced Institute of Life Sciences Jiangsu Engineering Research Center for MicroRNA Biology and Biotechnology Nanjing Jiangsu 210093 China; ^4^ Department of Lymphoma Tianjin Medical University Cancer Institute and Hospital National Clinical Research Center of Cancer Key Laboratory of Cancer Prevention and Therapy Tianjin 300060 China; ^5^ Nanjing Multicenter Biobank Biobank of Nanjing Drum Tower Hospital the Affiliated Hospital of Nanjing University Medical School Nanjing Jiangsu 210008 China

**Keywords:** apoptosis, esophageal cancer, miR‐1, proliferation, Src

## Abstract

Nonreceptor tyrosine kinase c‐Src, also known as Src, is a potent oncogene involved in a series of biological processes including cell growth, differentiation, and apoptosis; however, its expression pattern and function in esophageal cancer is poorly addressed. In this study, abnormal overexpression of Src protein was observed in esophageal cancer tissues, which fuelled the speculation that microRNA‐mediated posttranscriptional regulatory mechanism might be involved. Bioinformatic analyses were applied to identify miRNAs that could potentially target Src. miR‐1 was predicted and further validated as a direct repressor of Src. Moreover, we manipulated knockdown and overexpression experiment on TE‐1 and TE‐10 cells to demonstrate miR‐1 suppressed proliferation and promoted apoptosis in esophageal cancer cells by inhibiting Src. Taken together, this study underlines a negative regulatory mechanism in which miR‐1 serves as a suppressor of Src in esophageal cancer cells and may provide insights into novel therapeutic approaches for esophageal cancer.

## Introduction

Esophageal cancer is one of the most malignant tumors. It is estimated that esophageal cancer caused about 400,000 deaths in 2012 worldwide, ranking sixth among cancer‐related diseases 1. A large proportion of the patients are diagnosed with advanced esophageal cancer with dysphagia as the initial symptom, ripped off the chance to undergo surgical treatment 2. Even with the assistance of radiotherapy and concurrent definitive chemoradiotherapy, the 5‐year survival remains poor. Based on above, research on the molecular mechanism of esophageal cancer to provide thoughts on novel strategy for treatment is of great value.

Malfunctioning of oncogenes stands as the inherent characteristic of tumorigenesis, which can be brought about by genetic alterations mainly including gain of function mutation, amplifications, and epigenetic activation 3. Abnormal phosphorylation of nonreceptor tyrosine kinase c‐Src, hereafter referred to as Src, has long been considered as notoriously morbific for inducing carcinogenesis in the ways of proliferation, adhesion, angiogenesis, invasion, and apoptosis. More recently, disorganized amplification is proven to be another manner to cause dysregulation of Src in cancer. Amplification was found in up to 20% of the advanced human colorectal cancers. In contrast, artificial depletion of Src‐inhibited proliferation and invasion in triple‐negative breast cancer cell lines suggests the ectopic expression of Src is also in close connection with tumorigenesis 4. However, mechanism of Src dysregulation in other types of solid tumors is rarely addressed. Why does Src express in different manners and in what kind of mechanism does Src work during the initiation and progression of human cancers? These are ticklish questions remained to be answered.

MicroRNAs (miRNA) are 19~22‐nucleotide‐long noncoding RNAs that regulate gene expression in metazoans and plants 5, 6. miRNAs function by binding to their complementary sequences in the 3′ untranslated region of target gene transcripts, causing mRNA degradation and/or translational repression 7. The process of tumorigenesis has been considered to be accompanied by the dysregulation of a bunch of miRNAs. Theoretically, aberrant expression of miRNAs whose target genes are cancer‐related might initiate the changes in cell functions toward heterogeneity. On the positive side, utilizing miRNA to silence oncogenes that serve as hubs in carcinogenesis has set a trend for cancer care.

In this study, we found an inverse correlation between miR‐1 and Src, and verified that miR‐1 negatively regulates Src by complementarily binding to the 3′ untranslated region. As a consequence, miR‐1 promotes the proliferation and suppresses the apoptosis of esophageal cancer cells.

## Materials and Methods

### Human tissue

All methods and experimental protocols were approved by Nanjing University, and carried out in accordance with corresponding guidelines. Biospecimens were provided by Nanjing multicenter biobank, biobank of Nanjing Drum Tower Hospital, the Affiliated Hospital of Nanjing University Medical School, with consent of every donor, and normalized ethnic audit has been proceeded. Tissue specimens used in this study were frozen in liquid nitrogen immediately after dissection and stored at −80°C. Each tissue specimen was verified histologically and pathologically by the pathologist.

### Cell culture

The human esophageal cancer cell lines TE‐1 and TE‐10 were cultured in RPMI 1640 and DMEM, respectively, fortified with 10% fetal bovine serum (GIBCO, CA). All cells were incubated at 37°C in 5% CO_2_, water‐saturated atmosphere.

### Quantitative real‐time PCR(qRT‐PCR)

Total RNA from the frozen tissue specimens and cultured cells was isolated with TRIzol Reagent (Invitrogen, Carlsbad, CA) according to the manufacturer's instructions. Mature miRNA was quantified by virtue of Taqman microRNA probes (Applied Biosystems, Foster City, CA) according to the manufacturer's instructions.

To quantify Src and GAPDH mRNA, RT products including SYBR Green (TAKARA, China) and designed primers for Src and GAPDH were utilized. The primer sequence was as follows: Src (forward primer): 5′‐TGGCAAGATCACCAGACGG‐3′; Src (reverse primer): 5′‐GGCACCTTTCGTGGTCTCAC‐3′; GAPDH (forward primer): 5′‐CTGGGCTACACTGAGCACC‐3′; and GAPDH (reverse primer): 5′‐AAGTGGTCGTTGAGGGCAATG‐3′.

### Protein isolation and western blotting

The frozen tissue specimens and cultured cells were lysed in RIPA lysis buffer with freshly added protease inhibitor cocktail (Roche, Mannheim, Germany) and prepared for western blotting using an antibody against Src. Proteins were separated by 10% SDS‐PAGE before electro‐transferred to PVDF membrane (Roche, Indianapolis, IN). The membrane was incubated with primary antibodies after 1 h of blocking in 5% skim milk. The antibodies used were as follows: anti‐c‐Src (B‐12) (sc‐8056, Santa Cruz Biotechnology, CA) and anti‐GAPDH (sc‐365062). The signal was detected after the treatment of the SuperSignal West Pico chemiluminescence (Pierce, USA). Protein bands were quantified by the ImageJ software.

### Luciferase reporter assays

The full‐length human Src 3′‐UTR and the mutant Src 3′‐UTR were cloned into the p‐MIR‐reporter vector (Ambion, Austin, TX), respectively. TE‐1 cells were co‐transfected with the p‐MIR‐reporter vector, *β*‐galactosidase (*β*‐gal) expression vector (Ambion), and 10 pmol of miR‐1 mimic or scrambled negative control RNA. Relative luminescences were collected 24 h posttransfection using the luciferase assay kit (Promega, WI).

### Vector construction and siRNA interference assay

The Src overexpression was achieved by constructing vector (Germantown, MD) expressing the entire open reading frame (ORF) of human Src without miR‐1‐responsive 3′‐UTR. An empty vector was employed as the negative control.

RNA interference through the siRNA targeting human Src was designed and synthesized by GenePharma (Shanghai, China) to knockdown Src. The siRNA sequence was as follows: Src siRNA (sense): 5′‐CAGGCUGAGGAGUGGUAUUTT‐3′; Src siRNA (antisense): 5′‐AAUACCACUCCUCAGCCUGTT‐3′. Efficacy of the overexpression vector and the siRNA was identified by quantitative RT‐PCR and western blotting.

### Cell proliferation assay

The relative cell number was evaluated using the Cell Counting Kit‐8 (Dojindo) according to the manufacturer's instructions. Briefly, TE‐1/TE‐10 cells were seeded in 96‐well plate at the density of 3 × 10^4^ cells per well, counted at the indicated time points after transfected with miR‐1 mimic, Src siRNA, or Src overexpression vector. A total quantity of 10 *μ*L CCK‐8 liquid was added to each test well and the plate was incubated for 1 h at 37°C. The absorbance was detected at a wavelength of 450 nm.

### Apoptosis assays

The apoptosis of TE‐1 cells was evaluated by Annexin V‐FITC/PI staining kit (BD Biosciences, CA) according to the manufacturer's instructions. TE‐1/TE‐10 cells were cultured in a serum‐depleted environment for 24 h to induce apoptosis after transfected with miR‐1 mimic, Src siRNA, or Src overexpression vector. Different stages of apoptosis were distinguished by gating PI‐Annexin V‐positive cells on a fluorescence‐activated cell‐sorting (FACS) flow cytometer (BD Biosciences, CA).

### Statistical analysis

The data are presented in terms of means ± SE. *P *< 0.05 was considered statistically significant using Student's *t*‐test.

## Results

### The up‐regulation of Src protein level in esophageal cancer tissues

**Figure 1:**
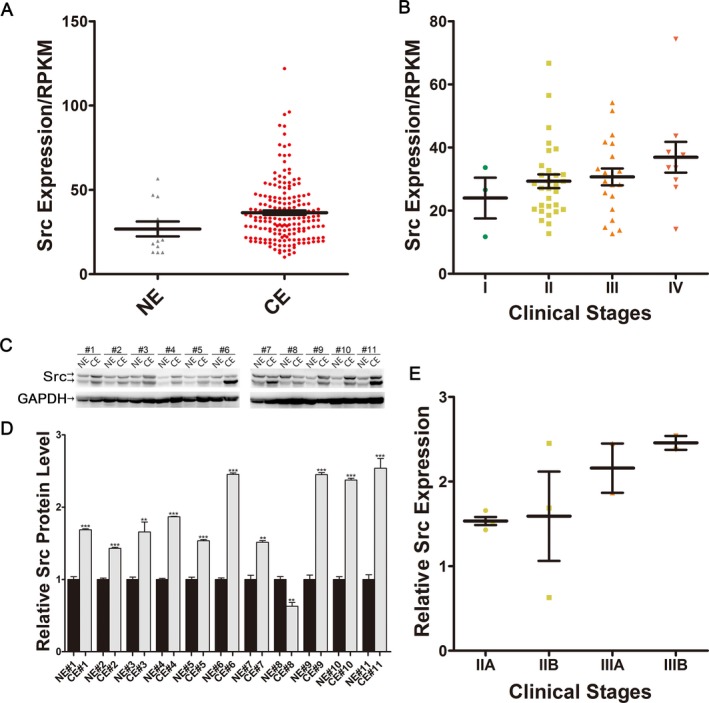
Expression patterns of Src in esophageal cancer tissues. (A) Quantification of Src mRNA expression in 183 human esophageal cancer tissues and 12 esophageal noncancerous tissues. (B) Quantification of Src mRNA expression of esophageal cancer tissues classified by clinical stages. (C and D) Src protein expression in 11 pairs of human esophageal cancer tissues and esophageal noncancerous tissues. GAPDH was used as a loading control. (C) Representative image; (D) Quantitative analysis. (E) Relative Src protein expression of esophageal cancer tissues classified by clinical stages.

We downloaded and reanalyzed the mRNA‐sequencing data of 183 esophageal cancer samples and 12 normal esophagus samples (the clinical information of these tissue specimens is listed in Table S1) from TCGA database. As shown in Figure 1A, Src expression was up‐regulated by 1.6‐fold in esophageal cancer tissues compared to normal esophagus. Sixty‐three esophageal cancer samples with traceable information were classified into four groups based upon clinical stage. The expression trend of Src went up with stages (Fig. 1B). Through quantifying Src protein expression levels via western blotting in 11 pairs of esophageal cancer and adjacent noncancerous tissues (the clinical information of these tissue specimens is listed in Table S2), 10 out of 11 pairs of tissues showed conspicuously higher levels of Src protein in cancer tissues compared to that in corresponding noncancerous tissues (Fig. 1C and D). Same Src expression trend in 11 pairs of esophageal tissues was observed (Fig. 1E).

### Prediction of conserved miR‐1 target site within Src 3′‐UTR

Given that the posttranscriptional regulation via microRNAs is one of the most common mechanisms seen in the dysregulation of oncogenes in solid tumors, we conjectured that miRNAs might also be involved in regulating Src expression in esophageal cancer.

**Figure 2:**
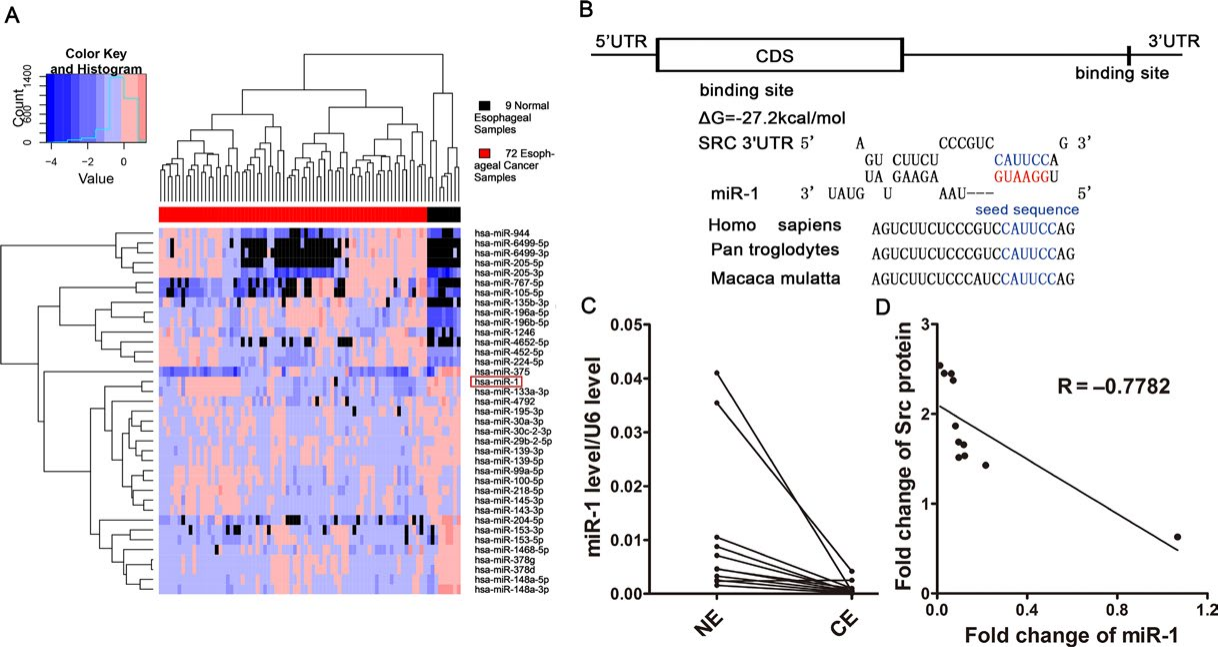
Prediction of miR‐1 binding site within Src 3′‐UTR. (A) Heat map of dramatically altered miRNAs from 72 esophageal cancer samples and nine normal esophagus. (B) Schematic description of the conjectural duplex formed by miR‐1 (bottom) and its binding site (top) within Src 3′‐UTR. The seed region of miR‐1 and the seed recognition site within Src 3′‐UTR are indicated in red and blue, respectively. All nucleotides in the seed recognition site were completely conserved among species. The predicted free energy value of the duplex is indicated. (C) Expression levels of miR‐1 in the same 11 pairs of CE and NE tissues. (D) Pearson's correlation scatter plot of the fold change of miR‐1 and Src protein in human esophageal cancer tissues.

In order to screen potential miRNAs that might participate in the tumorigenesis, we employed YM500 v2 to analysis the MiRs array profiling of 72 esophageal cancer samples and nine normal esophagus samples. Twenty miRNAs with most significant expression disparity were shown in the heat map (Fig. 2A), among which 14 miRNAs were down‐regulated in esophageal cancer (Table S3).

TargetScan 8 and RNAhydrid 9 were applied to narrow down the candidate miRNAs to two (miRNA‐1 and miRNA‐153‐3p), both of which were predicted to be able to target Src 3′‐UTR. However, luciferase reporter assay showed a less desirable affinity of miRNA‐153‐3p for Src 3′‐UTR on the human esophageal squamous cancer cell line TE‐1 (Fig. S1). Taken together, we predicted a miR‐1‐Src 3′‐UTR hybrid with the binding free energy value of −27.2  kcal/mol, as shown in Figure 2B. In addition, the cognate target of miR‐1 within Src 3′‐UTR was highly conserved across species, suggesting the biological functions of the match (Fig. 2B).

Negative feedbacks and distinct expression patterns in opposite directions are continuously seen between miRNAs and their targets 10, 11. Accordingly, we conducted quantitative RT‐PCR to determine miR‐1 expression levels in the same 11 pairs of esophageal cancer and adjacent noncancerous tissues. It was observed that esophageal cancerous tissues which had higher levels of Src, showed lower levels of miR‐1 (Fig. 2C). Interestingly, the expression of miR‐1 negatively correlated with Src protein level, as illustrated with Pearson's correlation scatter plots (Fig. 2D). Based upon the analytical prediction and the inverse expression patterns of miR‐1 and Src protein in esophageal cancer, we speculate that Src serves as a miR‐1 target.

### Src as the direct target of miR‐1

**Figure 3:**
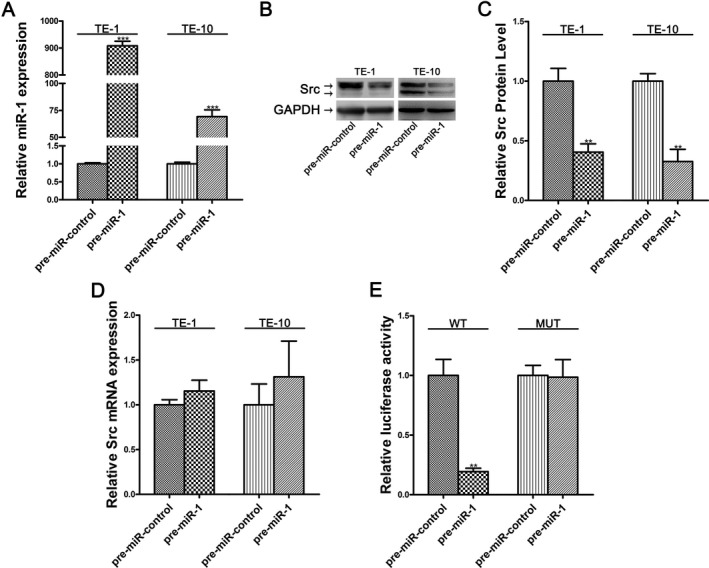
Src is a direct target of miR‐1. (A) Expression levels of miR‐1 in TE‐1/TE‐10 cells transfected with equal doses of the miR‐1 mimic (pre‐miR‐1) or scrambled negative control RNA (pre‐miR‐control). (B and C) Src protein expression in TE‐1/TE‐10 cells transfected with equal doses of the miR‐1 mimic or scrambled negative control RNA. GAPDH was used as a loading control. (B) Representative image; (C) Quantitative analysis. (D) Src mRNA levels in TE‐1/TE‐10 cells transfected with equal doses of the miR‐1 mimic or scrambled negative control RNA. (E) Relative luciferase activity of wild‐type (WT) and mutant (MUT) Src 3′‐UTR p‐MIR‐reporter vectors in TE‐1 cells transfected with the control mimic or miR‐1 mimic. Firefly luciferase values were normalized to *β*‐galactosidase activity.

The reciprocal relation between miR‐1 and Src was further confirmed through miR‐1 overexpression in TE‐1 and TE‐10 cells. Overexpression of miR‐1 was achieved by transfecting the cells with miR‐1 mimic (pre‐miR‐1), a chemically modified double‐strand RNA which mimicked the miR‐1 precursor. The efficiency of miR‐1 overexpression was shown in Figure 3A. Cellular miR‐1 levels increased by approximately 900‐fold and 70‐fold in TE‐1 and TE‐10 cells, respectively. As anticipated, Src protein was significantly down‐regulated in both cell lines after miR‐1 overexpression (Fig. 3B and C). To test whether miR‐1 modulated Src expression at the posttranscriptional level, we repeated the miR‐1 overexpression manipulation and determined the expression level of Src mRNA. No evidence was observed that overexpression of miR‐1 in TE‐1 and TE‐10 cells struck the homeostasis of Src mRNA (Fig. 3D). Results above strongly suggested that miR‐1 specifically repressed Src posttranscriptionally, reflecting the most classic modulatory mechanism for animal miRNAs.

We then performed the luciferase reporter assays to investigate whether the suppression of Src was induced by the complementary fixation as predicted. p‐MIR‐reporter vector carrying the full‐length Src 3′‐UTR in the downstream of the firefly luciferase was delivered into TE‐1 cells together with the gene *β*‐galactosidase (*β*‐gal) expression vector. Predictably, miR‐1 overexpression drastically reduced the luciferase activity (Fig. 3E). On the contrary, the luciferase activity of mutated p‐MIR‐reporter vector with introduced point mutations within the miR‐1 binding site remained steady under the overexpression manipulation (Fig. 3E). Taken together, these results substantiated that miR‐1 directly recognized and bound to the 3′‐UTR of the Src mRNA transcript to suppress Src in esophageal cancer cells.

### miR‐1 inhibits proliferation and promotes apoptosis of esophageal cancer cells by suppressing Src

**Figure 4:**
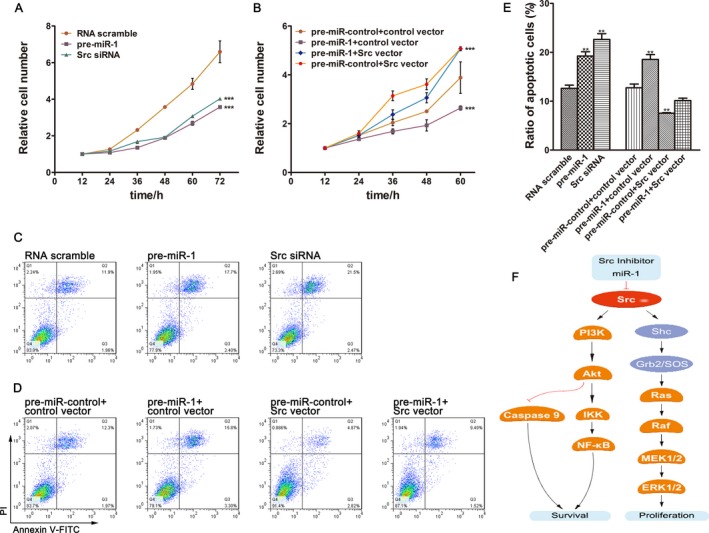
Roles of miR‐1 and Src in proliferation and apoptosis of esophageal cancer cells. (A) Growth curves of TE‐1 cells transfected with equal doses of the miR‐1 mimics or Src siRNA or scrambled control RNA. (B) Growth curves of TE‐1 cells transfected with the control mimic plus control vector or miR‐1 mimic plus control vector or miR‐1 mimic plus Src overexpression vector (Src vector) or control mimic plus Src overexpression vector. (C) Representative images of ratio of apoptotic TE‐1 cells transfected with equal doses of the miR‐1 mimics or Src siRNA or scrambled control RNA. (D) Representative images of ratio of apoptotic TE‐1 cells transfected with the control mimic plus control vector or miR‐1 mimic plus control vector or miR‐1 mimic plus Src overexpression vector or control mimic plus Src overexpression vector. (E) Quantitative analysis of the flow cytometry analysis of (C and D). (F) Model of miR‐1 enhances apoptosis and suppresses proliferation by targeting Src to restrain tumor growth in esophageal cancer.

To investigate the biological consequences of Src depletion, we tested TE‐1 cells treated with Src siRNA on cell proliferation via cell counting kit and on apoptosis via flow cytometric analysis. The efficiency of Src siRNA is demonstrated in Figure S2. As a result, knockdown of Src by siRNA lowered proliferation rate and raised apoptosis of TE‐1 cells (Fig. 4A and C). Then, we wondered whether the introduction of miR‐1 could achieve similar effects through suppressing Src expression. As expected, miR‐1 mimic produced the same effects as Src siRNA on proliferation and apoptosis of TE‐1 cells (Fig. 4A and C).

Moreover, to highlight the core roles of Src in modulating these biological processes, we simultaneously transfected the cells with miR‐1 mimic and vectors carrying the ORF of Src without the miR‐1‐responsive 3′‐UTR, expecting the effects of miR‐1 could be attenuated by Src overexpression. As shown in Figure 4B and D, TE‐1 cells co‐transfected with the Src overexpression vector and miR‐1 mimic exhibited elevated proliferation rate and decreased apoptosis compared to those treated with miR‐1 mimic alone. The results above served as evidence that miR‐1‐resistant Src expression could rescue the antiproliferation effect and inhibit the proapoptotic effect exerted by miR‐1.

## Discussion

Esophageal cancer is the eighth most common cancer and the sixth leading cause of cancer‐related deaths worldwide 12. Besides conventional surgery and chemotherapy, although efforts have been put in developing biological targeting drugs, prognosis of esophageal cancer remained poor with the 5‐year relative survival rate ranging from 10% to 25% 2, 13, 14. The plight appears to be attributable to the following reasons: Situation for esophageal cancer is similar to that for lung and pancreas cancers, where most cases are diagnosed at relatively advanced stages (to be specific, the proportions of localized, regional, and distant stage at diagnosis were 21%, 31%, and 38%, respectively) 15; Biological properties differ across two major histological types of esophageal cancer and expression levels of multiple related genes vary from case to case 16. Thus, good efficacy might not be yielded by single molecular target agent. In fact, targets for drugs in the treatment of esophageal cancer are mainly confined to VEGFR‐2, COX‐2, EGFR, mTOR, and HER‐2 17, 18. Accordingly, potent drugs directing at other crucial targets are drastically needed to use in combination with existing ones to modulate aberrantly activated signaling pathways in the broad and convoluted network.

Src stimulates signaling pathways that contribute to multiple cancer‐related process including survival and proliferation. As reported and illustrated with Figure 4F, Src activates PI3K/Akt pathway to negatively regulate Caspase 9 and simultaneously positively regulate NF‐kappa B while induces proliferation through MEK/ERK1/2 pathway 19, 20, 21. Accumulated evidence have demonstrated that the oncogenic Src is not only responsible for tumor progression but also inseparably involved in the resistance to anticancer drugs in conventional and targeted therapies 22. A study in which 278 HER2‐positive breast cancer cases were counted reported that active Src was positively correlated with trastuzumab resistance and even with shorter survival in patients at early stage with HER2/hormone receptor‐negative tumors treated with trastuzumab 23. In addition to frequent straggly phosphorylation, abnormal expressions of Src protein were also seen in neoplastic diseases. Elevated expression of Src protein was found to be related to invasiveness and metastasis in gastric tumors 24. Studies also asserted that overexpression of Src was observed in high‐grade leiomyosarcoma, making Src a potential valuable diagnostic marker for this soft tissue sarcoma 25. In this study, we showed ectopic overexpression of Src in esophageal tissues and observed that the progression of the esophageal cancer was closely associated with Src expression. Pro‐proliferative and antiapoptotic functions of Src in TE‐1 cells were further unraveled with silencing and overexpression manipulations.

Oncogenic miRNAs and tumor‐suppressive miRNAs are puissant molecules that regulate a range of cancer‐associated genes. Surprisingly, not only the presence or absence but also single nucleotide polymorphisms(SNPs) of these posttranscriptional regulators can contribute to tumorigenesis 26, 27, suggesting their mighty and irreplaceable roles played in this biological process. miR‐1 has long been regarded as a tumor‐suppressive miRNA and down‐regulations of miR‐1 were seen in a mount of malignancies, such as lung cancer, hepatocellular carcinoma, renal cell carcinoma, and colon cancer 28, 29. Consistent with our observation, miR‐1 was reported to correlate with advanced clinical stage in esophageal squamous cell carcinoma and served as a tumor suppressor by targeting LASP1 and TAGLN2 30, 31. Little wonder that we found the same expressional tendencies of miR‐1 in esophageal cancer tissues. Negative correlation between Src protein and miR‐1 was observed. Further experiments validated the targeting effect of miR‐1 on Src and the following cancer‐related functions of miR‐1 via Src in esophageal cancer cell line, which is concordant with the previous study that overexpression of miR‐1 reduces cell viability in HepG2 cells 32 and delays the growth rate of breast cancer stem cells, gastric and colorectal cancers 33, 34.

With the concept of genetically driven personalized medicine and the establishment of microRNA biology, we come to understand the significance and feasibility of antagonizing neoplastic diseases with the help of microRNA drugs. In this study, we delineated a regulatory network employing miR‐1 and Src to resettle biological functions including cell proliferation and apoptosis in esophageal cancer cell line. Unignorably,microRNAs carry the traits of multifunction and tissue specificity. For instance, miR‐1 expresses in a chamber‐specific manner during cardiogenesis and misregulation of miR‐1 causes heart defects. Down‐regulation of miR‐1 contributes to re‐expression of HCN2/HCN4 to start the remodeling process in hypertrophic hearts 35. Loss of miR‐1 also leads to the expression of GJA1 (connexin 43) and CACNA1C (Cav1.2) to generate calcium‐ and gap‐junction channels in myotonic dystrophy 36. In central nervous system, miR‐1 mediates the hypoxia‐induced cell injury by suppressing HSP‐70 with augmenting MMP and caspase‐3 activation at the same time 37. Despite the feasibility of microRNA drug been certified by translational studies on in vivo models and designing administration route for small molecular drugs had drawn much attention, challenge still lies in delivering the right amount of drugs to specific tissue to minimize side effect 38, 39.

In conclusion, our study indicated that Src is the key factor in modulating esophageal cancer process, and that miR‐1 is capable to negatively regulate the expression of Src and in turn restrain tumorigenesis in aspects of proliferation and apoptosis. These findings uncover the possibility to antagonize esophageal cancer with the new but old target.

## Conflicts of Interest

The authors declare no conflict of interest.

## Supporting information


**Figure S1**. Evaluation of the binding efficiency between miR‐153‐3p and Src 3’‐UTR.
**Figure S2.** Evaluation of knockdown and overexpression efficiency of Src in TE‐1 cells.
**Figure S3.** Effects of miR‐1 on proliferation and apoptosis of TE‐10 cells.
**Table S1.** Clinical information of 183 esophageal cancer specimens provided by TCGA database.
**Table S2.** Clinical information of esophageal cancer patients.
**Table S3.** Twenty microRNAs with the most significant expression disparity between esophageal cancer and normal esophagus.
