# Supplementary material for: miR‐1 suppresses the proliferation and promotes the apoptosis of esophageal carcinoma cells by targeting Src
Source: Cancer Med. 2017 Oct 16;6(12):2957–65. doi: 10.1002/cam4.1214 (PMC5727306; doi:10.1002/cam4.1214)
Supplement: Supplementary file 1 — Figure S1. Evaluation of the binding efficiency between miR‐153‐3p and Src 3’‐UTR. Figure S2. Evaluation of knockdown and overexpression efficiency of Src in TE‐1 cells. Figure S3. Effects of miR‐1 on proliferation and apoptosis of TE‐10 cells. Table S1. Clinical information of 183 esophageal cancer specimens provided by TCGA database. Table S2. Clinical information of esophageal cancer patients. Table S3. Twenty microRNAs with the most significant expression disparity between esophageal cancer and normal esophagus. [file CAM4-6-2957-s001.docx]

**Supplementary Materials**

**
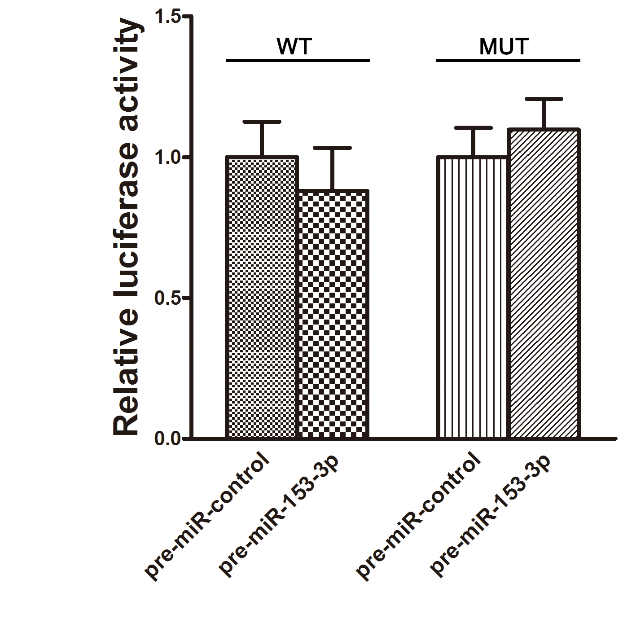
**

**Supplementary Figure 1. Evaluation of the binding efficiency between miR-153-3p and Src 3’-UTR.** Relative luciferase activity of wild-type (WT) and mutant (MUT) Src 3’-UTR p-MIR-reporter vectors in TE-1 cells transfected with control mimic or miR-153-3p mimic. Firefly luciferase values were normalized to β-galactosidase activity.

**
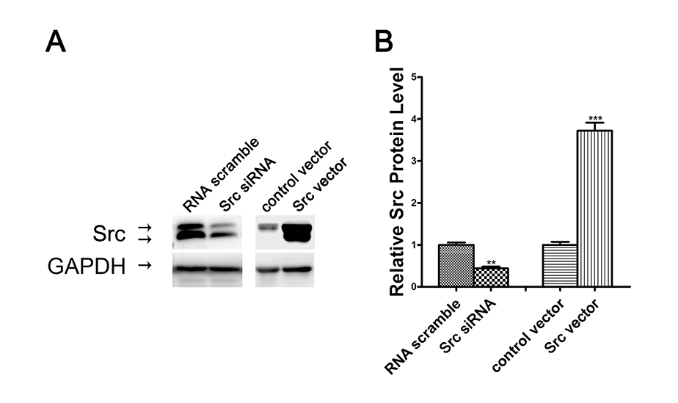
**

**Supplementary Figure 2. Evaluation of knockdown and overexpression efficiency of Src in TE-1 cells. (A and B)** Src protein level in TE-1 cells transfected with the scrambled negative control siRNA or Src siRNA, control vector or Src overexpression vector. A: representative image; B: quantitative analysis.

**
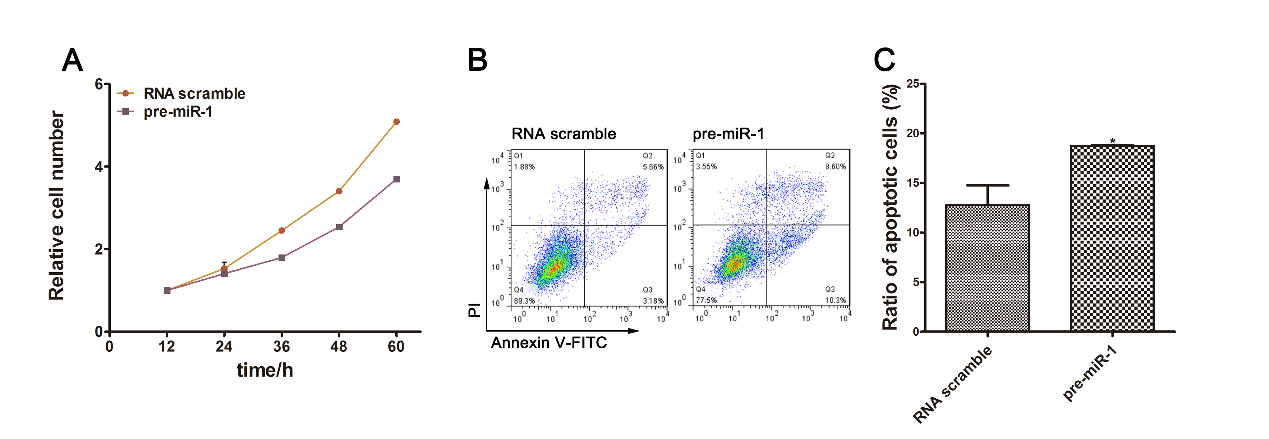
**

**Supplementary Figure 3. Effects of miR-1 on proliferation and apoptosis of TE-10 cells. (A)** Growth curves of TE-10 cells transfected with equal doses of the miR-1 mimics or scrambled control RNA. **(B)** Representative images of ratio of apoptotic TE-10 cells transfected with equal doses of the miR-1 mimics or scrambled control RNA. **(C)** Quantitative analysis of the flow-cytometry analysis of B.

**Supplementary Table 1. Clinical information of 183 esophageal cancer specimens provided by TCGA database.**

| **Clinical Stage** | **Age** | **Gender** | **Histological Type** | **Src Expression/RPKM** |
| --- | --- | --- | --- | --- |
| **NA** | **51** | **female** | **esophagus squamous cell carcinoma** | **30.5562** |
| **NA** | **54** | **male** | **esophagus squamous cell carcinoma** | **28.4859** |
| **NA** | **72** | **male** | **esophagus squamous cell carcinoma** | **20.4324** |
| **NA** | **53** | **male** | **esophagus squamous cell carcinoma** | **16.8859** |
| **NA** | **61** | **male** | **esophagus squamous cell carcinoma** | **32.6015** |
| **NA** | **62** | **male** | **esophagus squamous cell carcinoma** | **29.7122** |
| **NA** | **74** | **male** | **esophagus adenocarcinoma, nos** | **43.2662** |
| **NA** | **75** | **male** | **esophagus squamous cell carcinoma** | **34.5451** |
| **NA** | **77** | **male** | **esophagus adenocarcinoma, nos** | **53.7872** |
| **NA** | **90** | **male** | **esophagus squamous cell carcinoma** | **40.1445** |
| **NA** | **84** | **female** | **esophagus adenocarcinoma, nos** | **60.1197** |
| **NA** | **81** | **male** | **esophagus adenocarcinoma, nos** | **34.4669** |
| **NA** | **63** | **male** | **esophagus adenocarcinoma, nos** | **46.20845** |
| **NA** | **79** | **female** | **esophagus adenocarcinoma, nos** | **35.47285** |
| **NA** | **71** | **male** | **esophagus adenocarcinoma, nos** | **121.892** |
| **NA** | **79** | **male** | **esophagus adenocarcinoma, nos** | **19.2314** |
| **NA** | **70** | **female** | **esophagus adenocarcinoma, nos** | **45.1735** |
| **NA** | **54** | **female** | **esophagus squamous cell carcinoma** | **37.0102** |
| **NA** | **65** | **male** | **esophagus adenocarcinoma, nos** | **87.7653** |
| **NA** | **75** | **male** | **esophagus adenocarcinoma, nos** | **36.36385** |
| **NA** | **67** | **female** | **esophagus adenocarcinoma, nos** | **26.7373** |
| **NA** | **75** | **male** | **esophagus adenocarcinoma, nos** | **28.23585** |
| **NA** | **83** | **male** | **esophagus adenocarcinoma, nos** | **58.27035** |
| **NA** | **86** | **female** | **esophagus adenocarcinoma, nos** | **76.7306** |
| **NA** | **77** | **male** | **esophagus adenocarcinoma, nos** | **51.6592** |
| **NA** | **81** | **male** | **esophagus adenocarcinoma, nos** | **45.8796** |
| **NA** | **56** | **female** | **esophagus adenocarcinoma, nos** | **53.1761** |
| **NA** | **60** | **male** | **esophagus adenocarcinoma, nos** | **33.235** |
| **NA** | **48** | **male** | **esophagus squamous cell carcinoma** | **24.0155** |
| **NA** | **71** | **male** | **esophagus adenocarcinoma, nos** | **50.7957** |
| **NA** | **56** | **male** | **esophagus squamous cell carcinoma** | **10.1271** |
| **NA** | **65** | **male** | **esophagus squamous cell carcinoma** | **25.9248** |
| **NA** | **72** | **male** | **esophagus squamous cell carcinoma** | **16.3046** |
| **NA** | **58** | **male** | **esophagus adenocarcinoma, nos** | **46.5743** |
| **NA** | **69** | **female** | **esophagus squamous cell carcinoma** | **47.5308** |
| **NA** | **57** | **male** | **esophagus squamous cell carcinoma** | **20.9544** |
| **NA** | **54** | **male** | **esophagus squamous cell carcinoma** | **22.0877** |
| **NA** | **46** | **male** | **esophagus squamous cell carcinoma** | **33.1664** |
| **NA** | **60** | **male** | **esophagus squamous cell carcinoma** | **20.3341** |
| **NA** | **51** | **male** | **esophagus squamous cell carcinoma** | **21.0074** |
| **NA** | **56** | **male** | **esophagus adenocarcinoma, nos** | **83.1264** |
| **NA** | **51** | **male** | **esophagus squamous cell carcinoma** | **37.3898** |
| **NA** | **77** | **male** | **esophagus adenocarcinoma, nos** | **47.3817** |
| **NA** | **51** | **female** | **esophagus squamous cell carcinoma** | **21.3607** |
| **NA** | **49** | **male** | **esophagus squamous cell carcinoma** | **41.037** |
| **NA** | **50** | **male** | **esophagus squamous cell carcinoma** | **21.7789** |
| **NA** | **53** | **male** | **esophagus adenocarcinoma, nos** | **70.6296** |
| **NA** | **57** | **female** | **esophagus squamous cell carcinoma** | **28.7397** |
| **NA** | **61** | **male** | **esophagus adenocarcinoma, nos** | **31.1974** |
| **NA** | **54** | **male** | **esophagus squamous cell carcinoma** | **22.9139** |
| **NA** | **59** | **male** | **esophagus adenocarcinoma, nos** | **27.89795** |
| **NA** | **62** | **male** | **esophagus adenocarcinoma, nos** | **94.6663** |
| **NA** | **72** | **female** | **esophagus adenocarcinoma, nos** | **41.8036** |
| **NA** | **50** | **female** | **esophagus adenocarcinoma, nos** | **19.4173** |
| **NA** | **58** | **male** | **esophagus squamous cell carcinoma** | **18.5834** |
| **NA** | **59** | **male** | **esophagus squamous cell carcinoma** | **35.8206** |
| **NA** | **72** | **male** | **esophagus squamous cell carcinoma** | **45.7166** |
| **NA** | **50** | **male** | **esophagus squamous cell carcinoma** | **21.3496** |
| **NA** | **49** | **male** | **esophagus squamous cell carcinoma** | **33.3362** |
| **NA** | **84** | **male** | **esophagus squamous cell carcinoma** | **20.6076** |
| **NA** | **86** | **male** | **esophagus adenocarcinoma, nos** | **24.0054** |
| **NA** | **60** | **male** | **esophagus adenocarcinoma, nos** | **51.2665** |
| **NA** | **67** | **male** | **esophagus squamous cell carcinoma** | **20.4142** |
| **NA** | **76** | **male** | **esophagus adenocarcinoma, nos** | **75.8222** |
| **NA** | **70** | **female** | **esophagus squamous cell carcinoma** | **35.7116** |
| **NA** | **51** | **male** | **esophagus adenocarcinoma, nos** | **18.3578** |
| **NA** | **71** | **female** | **esophagus adenocarcinoma, nos** | **41.5841** |
| **NA** | **56** | **male** | **esophagus squamous cell carcinoma** | **26.3374** |
| **NA** | **52** | **male** | **esophagus squamous cell carcinoma** | **28.5754** |
| **NA** | **67** | **female** | **esophagus squamous cell carcinoma** | **28.5586** |
| **NA** | **53** | **male** | **esophagus squamous cell carcinoma** | **17.3255** |
| **NA** | **77** | **male** | **esophagus adenocarcinoma, nos** | **56.5906** |
| **NA** | **57** | **male** | **esophagus adenocarcinoma, nos** | **76.7665** |
| **NA** | **77** | **male** | **esophagus adenocarcinoma, nos** | **54.0515** |
| **NA** | **54** | **male** | **esophagus adenocarcinoma, nos** | **56.0875** |
| **NA** | **79** | **male** | **esophagus adenocarcinoma, nos** | **41.9489** |
| **NA** | **77** | **male** | **esophagus adenocarcinoma, nos** | **61.6353** |
| **NA** | **84** | **female** | **esophagus squamous cell carcinoma** | **19.3076** |
| **NA** | **56** | **male** | **esophagus adenocarcinoma, nos** | **45.1747** |
| **NA** | **84** | **female** | **esophagus adenocarcinoma, nos** | **35.8682** |
| **NA** | **81** | **male** | **esophagus adenocarcinoma, nos** | **39.1518** |
| **NA** | **71** | **male** | **esophagus squamous cell carcinoma** | **38.3033** |
| **NA** | **81** | **female** | **esophagus adenocarcinoma, nos** | **54.3348** |
| **NA** | **76** | **male** | **esophagus adenocarcinoma, nos** | **36.4934** |
| **NA** | **69** | **male** | **esophagus adenocarcinoma, nos** | **17.6477** |
| **NA** | **84** | **male** | **esophagus adenocarcinoma, nos** | **19.5092** |
| **NA** | **75** | **male** | **esophagus adenocarcinoma, nos** | **53.3895** |
| **NA** | **55** | **male** | **esophagus adenocarcinoma, nos** | **29.7266** |
| **NA** | **62** | **male** | **esophagus squamous cell carcinoma** | **31.9123** |
| **NA** | **54** | **male** | **esophagus squamous cell carcinoma** | **18.8815** |
| **NA** | **60** | **male** | **esophagus adenocarcinoma, nos** | **35.2757** |
| **NA** | **75** | **male** | **esophagus adenocarcinoma, nos** | **28.4165** |
| **NA** | **77** | **male** | **esophagus adenocarcinoma, nos** | **23.7891** |
| **NA** | **47** | **male** | **esophagus adenocarcinoma, nos** | **58.1681** |
| **NA** | **51** | **male** | **esophagus adenocarcinoma, nos** | **31.8822** |
| **NA** | **61** | **male** | **esophagus adenocarcinoma, nos** | **52.8881** |
| **NA** | **58** | **female** | **esophagus squamous cell carcinoma** | **18.4842** |
| **NA** | **36** | **male** | **esophagus squamous cell carcinoma** | **25.5445** |
| **NA** | **58** | **male** | **esophagus squamous cell carcinoma** | **36.0413** |
| **NA** | **65** | **male** | **esophagus adenocarcinoma, nos** | **36.4258** |
| **NA** | **42** | **male** | **esophagus squamous cell carcinoma** | **27.1417** |
| **NA** | **60** | **female** | **esophagus squamous cell carcinoma** | **14.152** |
| **NA** | **62** | **male** | **esophagus squamous cell carcinoma** | **37.7063** |
| **NA** | **70** | **male** | **esophagus squamous cell carcinoma** | **18.4818** |
| **NA** | **67** | **male** | **esophagus adenocarcinoma, nos** | **59.9082** |
| **NA** | **66** | **male** | **esophagus adenocarcinoma, nos** | **43.036** |
| **NA** | **44** | **male** | **esophagus adenocarcinoma, nos** | **42.3808** |
| **NA** | **68** | **male** | **esophagus adenocarcinoma, nos** | **25.4573** |
| **NA** | **57** | **male** | **esophagus adenocarcinoma, nos** | **45.4978** |
| **NA** | **43** | **male** | **esophagus adenocarcinoma, nos** | **44.8106** |
| **NA** | **74** | **male** | **esophagus adenocarcinoma, nos** | **33.6389** |
| **NA** | **53** | **male** | **esophagus adenocarcinoma, nos** | **88.2414** |
| **NA** | **70** | **male** | **esophagus adenocarcinoma, nos** | **52.2587** |
| **NA** | **58** | **male** | **esophagus adenocarcinoma, nos** | **57.4272** |
| **NA** | **80** | **male** | **esophagus adenocarcinoma, nos** | **21.2038** |
| **NA** | **80** | **male** | **esophagus adenocarcinoma, nos** | **70.4209** |
| **NA** | **53** | **male** | **esophagus squamous cell carcinoma** | **33.0684** |
| **NA** | **69** | **male** | **esophagus adenocarcinoma, nos** | **26.8667** |
| **NA** | **57** | **male** | **esophagus squamous cell carcinoma** | **38.8967** |
| **NA** | **67** | **male** | **esophagus squamous cell carcinoma** | **19.8779** |
| **stage ia** | **82** | **male** | **esophagus squamous cell carcinoma** | **11.727** |
| **stage ia** | **53** | **male** | **esophagus adenocarcinoma, nos** | **26.5762** |
| **stage ib** | **76** | **male** | **esophagus adenocarcinoma, nos** | **33.653** |
| **stage ii** | **65** | **male** | **esophagus squamous cell carcinoma** | **29.3395** |
| **stage iia** | **66** | **male** | **esophagus squamous cell carcinoma** | **20.4643** |
| **stage iia** | **44** | **male** | **esophagus squamous cell carcinoma** | **19.6767** |
| **stage iia** | **62** | **male** | **esophagus squamous cell carcinoma** | **15.805** |
| **stage iia** | **47** | **male** | **esophagus squamous cell carcinoma** | **19.8518** |
| **stage iia** | **71** | **male** | **esophagus squamous cell carcinoma** | **31.4532** |
| **stage iia** | **59** | **male** | **esophagus squamous cell carcinoma** | **31.2499** |
| **stage iia** | **62** | **male** | **esophagus squamous cell carcinoma** | **20.3943** |
| **stage iia** | **49** | **male** | **esophagus squamous cell carcinoma** | **27.1062** |
| **stage iia** | **44** | **male** | **esophagus squamous cell carcinoma** | **39.0857** |
| **stage iia** | **77** | **male** | **esophagus squamous cell carcinoma** | **16.861** |
| **stage iia** | **60** | **male** | **esophagus squamous cell carcinoma** | **29.2741** |
| **stage iia** | **57** | **male** | **esophagus squamous cell carcinoma** | **41.379** |
| **stage iia** | **49** | **male** | **esophagus squamous cell carcinoma** | **34.287** |
| **stage iia** | **52** | **male** | **esophagus squamous cell carcinoma** | **28.1467** |
| **stage iia** | **58** | **male** | **esophagus squamous cell carcinoma** | **21.852** |
| **stage iia** | **57** | **male** | **esophagus squamous cell carcinoma** | **12.7157** |
| **stage iia** | **49** | **male** | **esophagus squamous cell carcinoma** | **26.9899** |
| **stage iia** | **64** | **male** | **esophagus squamous cell carcinoma** | **21.7057** |
| **stage iia** | **44** | **female** | **esophagus squamous cell carcinoma** | **23.8882** |
| **stage iia** | **47** | **male** | **esophagus squamous cell carcinoma** | **21.4177** |
| **stage iia** | **56** | **male** | **esophagus adenocarcinoma, nos** | **46.3205** |
| **stage iia** | **60** | **male** | **esophagus adenocarcinoma, nos** | **66.7051** |
| **stage iib** | **50** | **male** | **esophagus squamous cell carcinoma** | **31.287** |
| **stage iib** | **69** | **female** | **esophagus squamous cell carcinoma** | **32.7624** |
| **stage iib** | **71** | **male** | **esophagus adenocarcinoma, nos** | **26.1248** |
| **stage iib** | **59** | **male** | **esophagus adenocarcinoma, nos** | **56.5319** |
| **stage iib** | **51** | **male** | **esophagus squamous cell carcinoma** | **39.5746** |
| **stage iib** | **49** | **male** | **esophagus squamous cell carcinoma** | **28.6274** |
| **stage iib** | **65** | **female** | **esophagus squamous cell carcinoma** | **18.5175** |
| **stage iii** | **81** | **male** | **esophagus adenocarcinoma, nos** | **24.59955** |
| **stage iii** | **46** | **male** | **esophagus squamous cell carcinoma** | **32.0544** |
| **stage iii** | **73** | **male** | **esophagus squamous cell carcinoma** | **12.7019** |
| **stage iii** | **61** | **male** | **esophagus squamous cell carcinoma** | **43.9877** |
| **stage iii** | **36** | **male** | **esophagus squamous cell carcinoma** | **20.3899** |
| **stage iii** | **46** | **male** | **esophagus squamous cell carcinoma** | **14.6649** |
| **stage iii** | **54** | **male** | **esophagus squamous cell carcinoma** | **28.2316** |
| **stage iii** | **70** | **male** | **esophagus squamous cell carcinoma** | **33.2258** |
| **stage iiia** | **58** | **male** | **esophagus squamous cell carcinoma** | **16.9337** |
| **stage iiia** | **74** | **female** | **esophagus adenocarcinoma, nos** | **31.4565** |
| **stage iiia** | **27** | **male** | **esophagus adenocarcinoma, nos** | **28.1353** |
| **stage iiia** | **64** | **male** | **esophagus adenocarcinoma, nos** | **41.7451** |
| **stage iiia** | **45** | **male** | **esophagus adenocarcinoma, nos** | **51.63455** |
| **stage iiia** | **57** | **male** | **esophagus squamous cell carcinoma** | **32.5167** |
| **stage iiia** | **60** | **male** | **esophagus squamous cell carcinoma** | **13.7824** |
| **stage iiia** | **72** | **male** | **esophagus adenocarcinoma, nos** | **54.1894** |
| **stage iiia** | **51** | **female** | **esophagus squamous cell carcinoma** | **25.6343** |
| **stage iiia** | **72** | **male** | **esophagus adenocarcinoma, nos** | **41.2906** |
| **stage iiib** | **57** | **male** | **esophagus squamous cell carcinoma** | **37.1749** |
| **stage iiic** | **58** | **male** | **esophagus squamous cell carcinoma** | **29.3081** |
| **stage iv** | **63** | **male** | **esophagus squamous cell carcinoma** | **36.4932** |
| **stage iv** | **58** | **male** | **esophagus adenocarcinoma, nos** | **38.5176** |
| **stage iv** | **42** | **male** | **esophagus adenocarcinoma, nos** | **37.6468** |
| **stage iv** | **68** | **male** | **esophagus adenocarcinoma, nos** | **33.509** |
| **stage iv** | **55** | **male** | **esophagus squamous cell carcinoma** | **43.64115** |
| **stage iv** | **51** | **male** | **esophagus squamous cell carcinoma** | **14.0837** |
| **stage iv** | **74** | **male** | **esophagus adenocarcinoma, nos** | **74.3663** |
| **stage iva** | **54** | **male** | **esophagus adenocarcinoma, nos** | **27.4104** |
| **stage iva** | **63** | **male** | **esophagus squamous cell carcinoma** | **33.6066** |
| **stage ivb** | **58** | **male** | **esophagus adenocarcinoma, nos** | **29.7385** |

**Supplementary Table 2. Clinical information of esophageal cancer patients.**

|  | **Age** | **Gender** | **Histological Type** | **Clinical Stage** |
| --- | --- | --- | --- | --- |
| **Case #1** | **51** | **Male** | **Squamous cell carcinoma** | **ⅡB(T2,N1,cM0)** |
| **Case #2** | **50** | **Male** | **Squamous cell carcinoma** | **ⅡA(T3,N0,cM0)** |
| **Case #3** | **64** | **Male** | **Squamous cell carcinoma** | **ⅡA(T3,N0,cM0)** |
| **Case #4** | **54** | **Female** | **Squamous cell carcinoma** | **ⅢA(T3, N1,cM0)** |
| **Case #5** | **58** | **Male** | **Squamous cell carcinoma** | **ⅡA(T3,N0,cM0)** |
| **Case #6** | **74** | **Male** | **Squamous cell carcinoma** | **ⅡB(T3,N0,cM0)** |
| **Case #7** | **60** | **Male** | **Squamous cell carcinoma** | **ⅡA(T3,N0,cM0)** |
| **Case #8** | **71** | **Male** | **Squamous cell carcinoma** | **ⅡB(T2,N1,cM0)** |
| **Case #9** | **77** | **Male** | **Squamous cell carcinoma** | **ⅢA(T3,N1,cM0)** |
| **Case #10** | **65** | **Male** | **Squamous cell carcinoma** | **ⅢB(T3,N2,cM0)** |
| **Case #11** | **70** | **Male** | **Squamous cell carcinoma** | **ⅢB(T3,N2,cM0)** |

**Supplementary Table 3.** **Twenty microRNAs with the most significant expression disparity between esophageal cancer and normal esophagus.**

| **miR Name** | **Mean expression** | **9 Normal Eso-**  **phageal Samples** | **72 Esophageal Cancer Samples** | **foldChange** | **log2FoldChange** | **pval** | **padj** |
| --- | --- | --- | --- | --- | --- | --- | --- |
|  |  | **Mean expression** | **Mean expression** |  |  |  |  |
| **hsa-miR-1** | **148.1362** | **611.9222** | **90.16298** | **0.14734** | **-2.76274** | **0.00019** | **0.01221** |
| **hsa-miR-100-5p** | **7355.858** | **22112.33** | **5511.299** | **0.24924** | **-2.00439** | **2.00E-05** | **0.00241** |
| **hsa-miR-105-5p** | **191.6205** | **2.337** | **215.2809** | **92.11855** | **6.52542** | **6.00E-05** | **0.00487** |
| **hsa-miR-1246** | **53.77469** | **1.35073** | **60.32769** | **44.66315** | **5.48101** | **0.00013** | **0.00965** |
| **hsa-miR-133a-3p** | **190.5328** | **867.0587** | **105.967** | **0.12221** | **-3.03251** | **0.00056** | **0.0286** |
| **hsa-miR-135b-3p** | **8.34439** | **0.28075** | **9.35235** | **33.31242** | **5.05799** | **4.00E-05** | **0.00355** |
| **hsa-miR-139-3p** | **29.5039** | **93.36637** | **21.5211** | **0.2305** | **-2.11715** | **1.00E-05** | **0.00189** |
| **hsa-miR-139-5p** | **187.1093** | **636.3145** | **130.9587** | **0.20581** | **-2.28063** | **0** | **0.00067** |
| **hsa-miR-143-3p** | **871227.2** | **3139317** | **587716** | **0.18721** | **-2.41726** | **0.00029** | **0.01699** |
| **hsa-miR-145-3p** | **153.0772** | **473.8895** | **112.9756** | **0.2384** | **-2.06854** | **0.00022** | **0.01355** |
| **hsa-miR-1468-5p** | **17.1474** | **57.64788** | **12.08484** | **0.20963** | **-2.25407** | **4.00E-05** | **0.00355** |
| **hsa-miR-148a-3p** | **84456.25** | **445883.6** | **39277.84** | **0.08809** | **-3.50488** | **0** | **0** |
| **hsa-miR-148a-5p** | **85.71733** | **300.4705** | **58.87318** | **0.19594** | **-2.35154** | **1.00E-05** | **0.0015** |
| **hsa-miR-153-3p** | **8.42196** | **38.31652** | **4.68514** | **0.12227** | **-3.0318** | **0** | **9.00E-05** |
| **hsa-miR-153-5p** | **49.59546** | **211.1938** | **29.39567** | **0.13919** | **-2.84489** | **2.00E-05** | **0.00213** |
| **hsa-miR-195-3p** | **11.27556** | **32.17639** | **8.66296** | **0.26923** | **-1.89307** | **0.00013** | **0.00957** |
| **hsa-miR-196a-5p** | **471.5389** | **8.03782** | **529.4765** | **65.87316** | **6.04162** | **0** | **0** |
| **hsa-miR-196b-5p** | **366.6016** | **6.63259** | **411.5977** | **62.05688** | **5.95552** | **0** | **0** |
| **hsa-miR-204-5p** | **38.64703** | **274.4334** | **9.17373** | **0.03343** | **-4.9028** | **0** | **0** |
| **hsa-miR-205-3p** | **7.12693** | **0** | **8.01779** | **0** | **0** | **0** | **0.00027** |
